# Clinical Interventions for Hyperacusis in Adults: A Scoping Review to Assess the Current Position and Determine Priorities for Research

**DOI:** 10.1155/2017/2723715

**Published:** 2017-10-09

**Authors:** Kathryn Fackrell, Iskra Potgieter, Giriraj S. Shekhawat, David M. Baguley, Magdalena Sereda, Derek J. Hoare

**Affiliations:** ^1^NIHR Nottingham Biomedical Research Centre, Ropewalk House, Nottingham, UK; ^2^Otology and Hearing Group, Division of Clinical Neuroscience, School of Medicine, University of Nottingham, Nottingham, UK; ^3^Health Systems and Audiology, The University of Auckland, Auckland, New Zealand; ^4^Tinnitus Research Initiative, Regensburg, Germany

## Abstract

**Background:**

There is no universally accepted definition for hyperacusis, but in general it is characterised by decreased sound tolerance to ordinary environmental sounds. Despite hyperacusis being prevalent and having significant clinical implications, much remains unknown about current management strategies.

**Purpose:**

To establish the current position of research on hyperacusis and identify research gaps to direct future research.

**Design and Sample:**

Using an established methodological framework, electronic and manual searches of databases and journals identified 43 records that met our inclusion criteria. Incorporating content and thematic analysis approaches, the definitions of hyperacusis, management strategies, and outcome measures were catalogued.

**Results:**

Only 67% of the studies provided a definition of hyperacusis, such as “reduced tolerance” or “oversensitivity to sound.” Assessments and outcome measures included Loudness Discomfort Levels, the Hyperacusis Questionnaire, and Tinnitus Retraining Therapy (TRT) interview. Management strategies reported were Cognitive Behavioural Therapy, TRT, devices, pharmacological therapy, and surgery.

**Conclusions:**

Management strategies were typically evaluated in patients reporting hyperacusis as a secondary complaint or as part of a symptom set. As such the outcomes reported only provided an indication of their effectiveness for hyperacusis. Randomised Controlled Trials are needed to evaluate the effectiveness of management strategies for patients experiencing hyperacusis.

## 1. Introduction

Hyperacusis is the perception of everyday environmental sound as being overwhelmingly loud or intense. Other terminology in use includes reduced, decreased, or collapsed sound tolerance. It differs from phonophobia which is an episodic sound intolerance experienced by some people during migraine attacks, sometimes associated with other sensory sensitivities, and which abates as the attack recedes [1]. It can also be differentiated from misophonia which is an acquired aversive reaction to specific human generated sounds such as eating sound or breathing, the response being characterised by anger and sometimes rage [2, 3].

As with other subjective symptoms, data on the prevalence of hyperacusis is strongly influenced by how the enquiry about the symptom was worded. In children and adolescents, the variability in wording of the enquiry in prevalence studies has been found to be so great as to render comparison across studies to be meaningless [4]. In a study of 7096 11-year-old children in the UK, 3.7% answered affirmatively to the question “do you ever experience oversensitivity or distress to particular sounds?” [5]. In adults, variability of the questions asked across studies of hyperacusis also significantly impacts on the ability to identify prevalence figures. Using the question “Do you consider yourself sensitive to everyday sounds?,” Andersson and colleagues [6] found that of 1,174 adults that answered the question via either the Internet or post, 8.6% (95% CI = 7.0–10.0) responded affirmatively. Another Swedish study [7] in adults asked 3406 participants, “Do you have a hard time tolerating everyday sounds that you believe most other people can tolerate?” and found 9.2% responded affirmatively, with 1.9% reporting that they had been diagnosed with hyperacusis by a physician.

There is an apparent association between hyperacusis and tinnitus, with 86% of adult patients with a primary complaint of hyperacusis experience tinnitus [8] and 40% of patients with a primary complaint of tinnitus experience hyperacusis as well [9]. Many people with troublesome hyperacusis have normal or age appropriate hearing thresholds, but cochlear hearing loss has also been reported [10]. Medical conditions associated with hyperacusis include closed head injury, depression, posttraumatic stress syndrome, Williams syndrome, and pain syndromes such as fibromyalgia [1]. The physiological mechanisms that underpin hyperacusis are not well understood, and there is no compelling animal model. A consensus is emerging in the auditory neuroscience literature that hyperacusis may be associated with a sustained and persistent increase in central auditory gain [11].

A framework with which to categorise patients with hyperacusis has been proposed [12]. The subtypes consisted of hyperacusis that is characterised by loudness, annoyance, fear, or pain. This schema was not based upon empirical data and may serve to illustrate the various characteristics that can define the lived experience of hyperacusis, rather than to support diagnosis or treatment. Regardless, hyperacusis is a complex symptom which can have negative effects on daily functioning such as hearing, sleep, concentration, and emotional well-being that can vary daily and between individuals. Management, therefore, can be complex with approaches taken including cognitive behaviour therapy, Tinnitus Retraining Therapy, or sound therapy [13]. Complex interventions such as these need to be developed and evaluated in a systematic way to have confidence in the effectiveness of the intervention for the given population [14].

The purpose of this scoping review is to identify research gaps in existing literature and elements that should underpin the design of any new studies. The aim of this scoping review is to consider the current position of research on hyperacusis in adults. Specifically, the objective here is to identify

- how hyperacusis is currently defined in research studies,
- How it is currently measured (i.e., what measures are used for diagnosis and outcome and are they adequate?),
- What the level of evidence is for current management options (i.e., what has been previously studied and to what extent?).

## 2. Materials and Methods

This review is reported according to the methodological framework developed by Arksey and O'Malley [15] using the six-stage process. In this process (1) the purpose and research questions were defined, (2) relevant studies were identified, (3) studies were selected using an iterative approach through title, abstract, and full-text screening by three members of team (KF, IP, and GSS), (4) data were extracted and charted by two members of the team (KF, IP), (5) the results were collated, summarised, and reported, and (6) two clinical experts, who were not involved in the data extraction or collating and summarising results, were consulted and reviewed the findings.

### 2.1. Eligibility Criteria

To be included, records were required to report studies in which adults (≥18 years old) reported hyperacusis as a primary complaint or secondary complaint or as part of a symptom set. Records were included where management strategies (i.e., interventions) were tested to address hyperacusis. Records were eligible from Randomised Controlled Trials (RCTs), nonrandomised control trials, cohort studies, case series, and case studies. Review articles including systematic reviews, epidemiology articles, and any sources reporting personal/expert opinions were excluded. No records were excluded based on controls used, outcomes reached, timing, setting, or study design. Records were excluded for studies reporting misophonia, phonophobia, and loudness recruitment.

All included records were published in the English language. Where multiple eligible unique records pertaining to a single trial were identified, the record that was published first was included and any secondary analyses of the data were excluded.

### 2.2. Search Strategy

The search strategy followed a recommended three-step approach [16, 17]. In Step 1, to test keywords and search terms, a limited search in PsycINFO and Embase was conducted, checking the availability of relevant titles and abstracts. This allowed us to develop search term combinations to use across a wider search in step two. The search strategy included hyperacusis and terms for identifying research studies, such as intervention, therapy, treatment, management, assessment, outcome, and diagnoses (diagnostic) with narrow terms such as measure and test (Table 1). In Step 2, to identify relevant research studies, electronic databases of peer reviewed journals were searched in the Cochrane Ear, Nose and Throat Disorders Group Trials Register; the Cochrane Central Register of Controlled Trials (CENTRAL); PubMed; Embase; PsycINFO; Scopus; Cumulative Index to Nursing and Allied Health Literature (CINAHL); Web of Science; the International Standard Randomised Controlled Trial Number (ISRCTN) registry; ClinicalTrials.gov; the International Clinical Trials Registry Platform (ICTRP); and Google Scholar. Specific search term strategies were applied in each search engine, searching article topics, titles, abstracts, and keywords. Where possible, filters were applied to retrieve articles in the English language and using human participants only. There was no restriction in the search period as we wanted to include all available research up until the present time.

In Step 3, to seek further eligible documents for inclusion, we performed manual searches of the reference lists of any relevant review articles which had hyperacusis in the title. In addition, manual searches of the most common journals (determined using the interquartile rule for outliers) in which eligible records had been sourced were conducted. The final manual search was conducted in April 2017.

### 2.3. Study Selection

Articles identified through electronic and manual searches were exported with citations, title, and abstract into Endnote where duplicates were removed. Search records were screened independently by two researchers out of a team of three (KF, IP, and GSS), first screening by title and abstract and then by full text. When disagreements regarding the inclusion or exclusion of any given record arose, the two researchers discussed their rationale until agreement was reached or a third researcher (DH) was consulted to reach a majority decision.

### 2.4. Data Extraction

A data extraction form was developed and piloted on two included records and was subsequently modified following team discussions. Data from each article were extracted by two researchers (KF and IP). Data were extracted on study characteristics, definition of hyperacusis, assessment measures used, and interventions (Box 1).

## 3. Results

### 3.1. Study Selection

**Figure 1:**
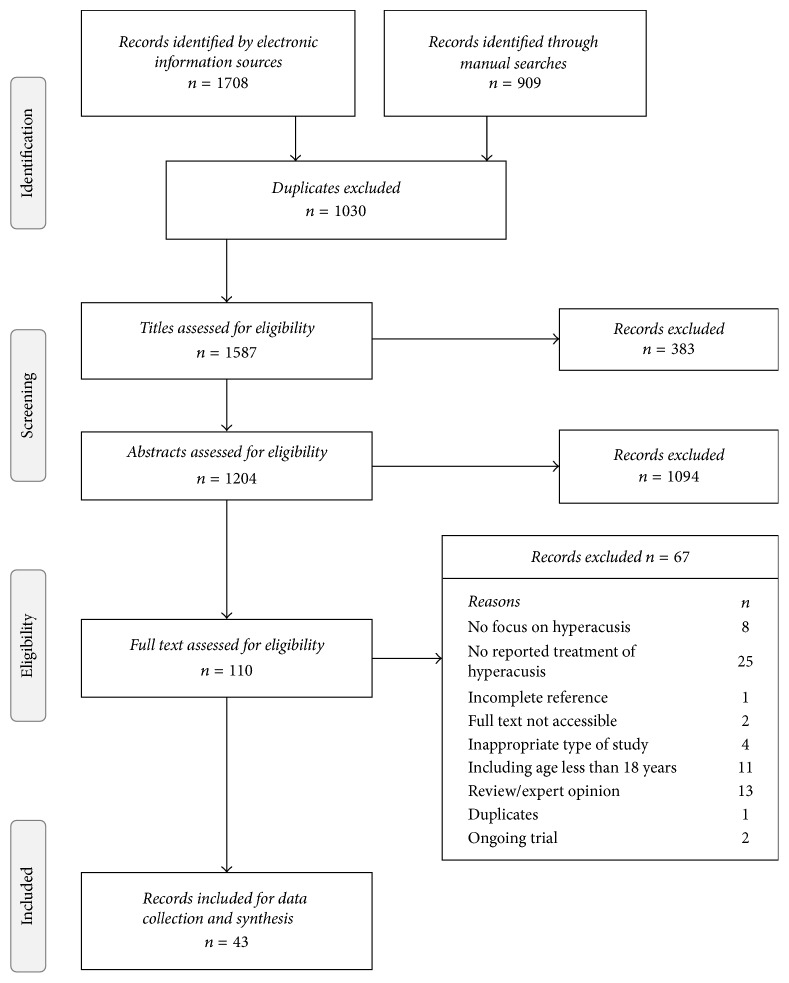
Flow chart of stages of study selection process.

Figure 1 illustrates the flow of study records identified, screened, included, and excluded (with reasons for exclusion). Electronic searches yielded an initial set of 1708 records. Duplicates were removed and of the remaining 792 records, 710 were excluded because the title and abstract indicated that the article did not fit our eligibility criteria. Most commonly the studies excluded did not focus on hyperacusis or did not report treatment of hyperacusis. Manual searches of included records in review articles identified a further 27 potential articles which were subjected to full-text screening. Manual searches in the selected journals identified one additional eligible record. Sixty-seven records were excluded at the full-text screening stage. Commonly, this was because the record did not report on the treatment or management of hyperacusis or was based on expert opinion or a review of the literature. For 1 record the reference was incomplete and could not be found. Full-text records could not be located for a further 2 records. None of these records could be traced, regardless of support from the University of Nottingham librarian. The electronic and manual searches created a final list of 43 eligible full-text records for data collection.

### 3.2. Study Characteristics

Of the 43 included full-text articles, there were 34 journal articles, six conference papers, and three book chapters. Articles were published from 1984, with the majority published after 2001 and the most recent being published in 2016. Records were predominantly reporting studies from the USA, UK, Australia, India, South Korea, and European countries. Of the included records, 23 were case studies [19, 25–27, 34–38, 40–43, 49–58], seven were cohort studies [28, 29, 39, 44, 46, 59, 60], five were RCTs [18, 20, 21, 30, 47], four were Nonrandomised Controlled Trials [22, 23, 31, 48], and four were retrospective studies [24, 32, 33, 45].

### 3.3. Population Characteristics

Of the 43 included records, 16 studies [18, 19, 21–25, 38, 39, 41–43, 55–57, 60] included patients reporting hyperacusis as a primary complaint. Of these studies, nine (53%) were case studies and only two (12%) were RCTs (Table 2). Equally, 16 studies [34–37, 40, 44, 45, 47, 49–54, 58, 59] included patients reporting hyperacusis as part of a set of symptoms, the most common of which were tinnitus, hearing loss, vertigo, or aural fullness. The remaining 11 studies included patients reporting hyperacusis as a secondary complaint to tinnitus [20, 26–28, 30–33, 48] and/or hearing loss [29, 46]. Duration of the participants hyperacusis was not reported in 29 studies [20–26, 28–33, 38, 39, 42–48, 50–53, 56, 57, 59]. For the remaining 14 studies [18, 19, 27, 34–37, 40, 41, 49, 54, 55, 58, 60], the duration of hyperacusis reported ranged from 6 weeks in a surgical case study [55] to 27 years in an “acoustic training” case study [40].

### 3.4. Current Definitions for Hyperacusis

**Figure 2:**
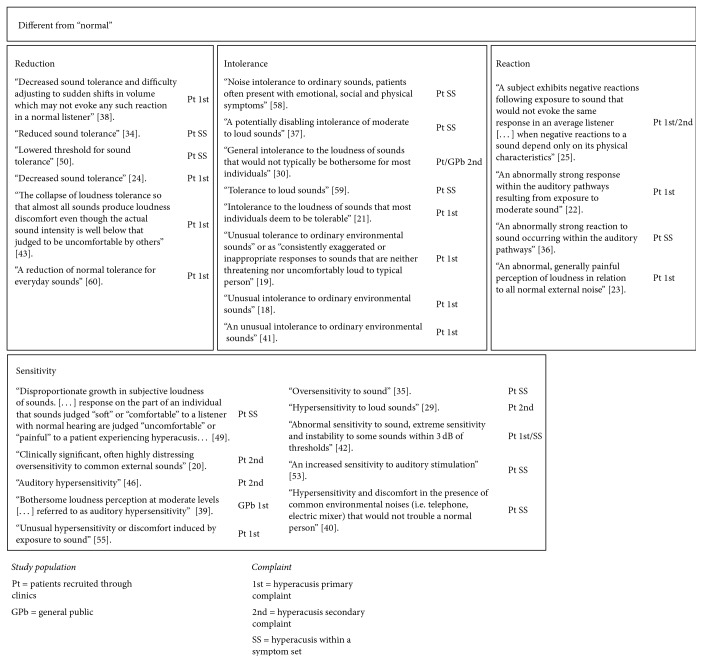
The four themes and underlying theme to emerge from the content of the working definitions of hyperacusis reported in the included records.

Fourteen studies [26–28, 31–33, 44, 45, 47, 48, 51, 52, 54, 57] did not provide a working definition of hyperacusis. Across the remaining studies, common terminologies were used to define hyperacusis, with four main themes identified (Figure 2).

One theme that emerged from the definitions focused on* “reductions”* in sound tolerance [24, 34, 38, 43, 50, 60]. Authors described hyperacusis as “a reduction of normal tolerance for everyday sounds” [38], “decreased sound tolerance” [24], “lowered threshold for sound tolerance” [50], or “the collapse of loudness tolerance” [43]. In contrast to this, other studies emphasised the* “increased sensitivity”* to sound [20, 29, 35, 39, 40, 42, 46, 53, 55]. This theme included descriptors such as “hypersensitivity,” “oversensitivity,” or “abnormal sensitivity” to define the degree of tolerance to sounds (Figure 2). One author used descriptors that indicated increased loudness, “a disproportionate growth in subjective loudness of sounds” [49]. Other definitions referred to* “intolerance”* to sounds that had been deemed as ordinary [18, 19, 21, 30, 37, 41, 58, 59]. Authors referred to “noise intolerance to ordinary sounds” [58], “unusual intolerance” [18, 19], or “intolerance to the loudness of sounds that most individuals deem to be tolerable” [21]. The last theme to emerge included definitions that referred to the physical or emotional reaction to the sound, such as “subject exhibits negative reactions” [25], “abnormal, generally painful perception of loudness” [23], or the “response or reaction from the auditory cortex” [22, 36].

Underlying these themes, throughout, was the common concept of the tolerance to sound being different from “normal.” Definitions referred to sound tolerance of “normal listeners” [38], “a normal person” [40], “a typical person” [19], or “others” [43] in comparison to sound tolerance of patients with hyperacusis. For example, Formby et al. [30] described hyperacusis as a “general intolerance to the loudness of sounds that would not typically be bothersome for most individuals.” In some cases, the response to “ordinary” or “everyday” environmental sounds was described as “unusual” [18, 19, 55] or “abnormal” [22, 23, 36, 42], and in one case the response was described as “exaggerated or inappropriate” [19].

Across the themes reductions, sensitivity, and intolerance, some authors noted within their working definitions the overt physical and emotional responses evoked by the perception of sounds. These authors highlighted the “bothersome” [30, 39], “distressing” [20], or “disabling” [37] nature of the sounds, the “discomfort” [19, 40, 43, 49, 55] or “pain” [23, 49] caused by the perception of sound, or the negative reaction to exposure to sound [25]. In one case, Silverstein et al. [58] listed associated symptoms of “emotional, social and physical” reactions to hyperacusis within their working definition.

### 3.5. Assessment Tools Used for Diagnosis and Outcome

In six studies (14%), the assessment tools used to quantify or diagnosis hyperacusis were not stated [20, 21, 39, 43, 47, 59]. Sixteen studies used clinical interviews (history and examination) to assess patient symptoms [28, 29, 35, 36, 41, 42, 46, 49–57]. Most of these studies were case series and reports, with some implying that interviews were used but not explicitly stating this.

Of the remaining 21 studies [18, 19, 22–27, 30–34, 37, 38, 40, 44, 45, 48, 58, 60], audiometric measures (Loudness Discomfort Levels (LDLs), Maximum Comfort Levels (MCLs), and Dynamic Range (DR)) and patient self-reported measurement tools (Baltimore questionnaire (VAS) [61], Sound Hypersensitivity Questionnaire [62], Hyperacusis Questionnaire (HQ) [63], Hyperacusis test (12-item) [38], a non-validated Hyperacusis Questionnaire adapted from Geraüschüberempfindlichkeit (GÜF [64]) [60], and Tinnitus Retraining Therapy (TRT) assessment interview [65]) were reported to assess hyperacusis. Most commonly reported were LDLs, the HQ and TRT assessment interview. The six studies [18, 22, 24, 30, 31, 37] that reported using LDLs varied in the dB values used to quantify hyperacusis. Gold et al. [22] and Hazell et al. [24] specified that average LDLs should be below 100 dB HL for 1, 2, 3, and 8 kHz in both ears (pure tones), whilst Jüris et al. [18] and Formby et al. [30] specified that LDLs using pure tones should be ≤90 dB HL in at least one ear at the frequencies of 0.5, 1, and 2 kHz or from at least two frequencies in the range 0.5 kHz to 4 kHz, respectively. In contrast, McKinney et al. [31] specified less commonly used LDLs using pure tones of less than 88.39 dB SPL and 81.24 dB SPL to classify individuals as having hyperacusis with normal hearing and hearing loss, respectively. Ruth and Hamill-Ruth [37] did not specify any criteria for LDLs. A similar case was found across the four studies reporting the HQ [19, 45, 48, 58]. Only one study [19] specifically identified a value on the HQ that would diagnose hyperacusis (36 points). Seven studies used the TRT assessment interview to classify patients into one of five categories associated with tinnitus and hyperacusis [24–27, 32–34]. Of these studies, only Hazell et al. [24] explicitly referred to categories 3 and 4 (hyperacusis present).

The most commonly specified posttreatment outcome measures were LDLs and the HQ (Tables 2–4). Nineteen studies reported increases in LDLs after treatment as either a primary [18, 21–25, 30, 31, 33, 34, 42, 47, 60] or secondary outcome [19, 28, 36, 38, 39, 58]. Six studies reported changes in HQ scores as the primary outcome [19, 39, 45, 46, 48, 58] or secondary outcome measure [18]. Of the 43 included records, 13 case studies [26, 35–37, 41, 43, 49, 50, 52–57] and two cohort studies [29, 59] relied on patient self-reported change in hyperacusis through clinical assessment at follow-up. One study [20] did not state a hyperacusis-specific outcome measure, only reporting the Tinnitus Questionnaire [66] as an outcome measure. The remaining studies reported a variety of different outcome measures, ranging from single item visual analogue scales to multi-item hyperacusis-specific questionnaires such as Multiple Activity Scale for Hyperacusis (MASH) [67] and Sound Hypersensitivity Questionnaire [62].

There is reasonable body of evidence on the development and reliability of the HQ as a diagnostic tool [63, 68], but the validity and reliability of the HQ as an outcome measure are yet to be fully examined. Questions have been raised on the appropriateness of the items in the questionnaire and the need of validation in a population with a primary complaint of hyperacusis is known [68]. Evidence for the reliability of LDLs is variable [69, 70] with reliability depending on a number of factors including instructions given to patients [71] and choice of sound stimuli [8]. Importantly, there are inconsistencies in the relationship between LDLs and self-report sound tolerance, with LDLs (using pure tones or speech sounds) often failing to reliably relate to self-reports of tolerance sounds in daily life [72].

### 3.6. Current Management Options for Hyperacusis

All 43 included records reported potential treatment benefits for hyperacusis (Tables 2–4). However, only nineteen studies (44%) sought to evaluate interventions or management strategies specifically aimed at reducing hyperacusis [18, 19, 21–25, 35, 38–43, 49, 56–58, 60]. For the most part, the studies explored interventions that were primarily aimed at reducing tinnitus. Management strategies explored were Cognitive Behavioural Therapy (CBT), TRT, counselling, devices, pharmacological therapy, and surgery.

### 3.7. Cognitive Behavioural Therapy

Three studies explored the potential benefits of CBT [18–20] (Table 2). Jüris et al. [18] reported an RCT investigating the benefits of CBT for patients experiencing hyperacusis. Treatment comprised general CBT principles that were aimed to educate, target overt emotional reactions to sounds though graded exposure to sounds, reduce stress though relaxation, and provide patients with the tools to manage more difficult situations and restart activities (behavioural activation). After treatment, patients in the CBT group showed a significant reduction in hyperacusis severity as assessed by the HQ and an increase in LDLs from baseline, compared to the waiting list group. Only small effects were observed for quality of life and depression, and symptoms of anxiety were unchanged. Fioretti et al. [19] reported a case study in which a patient with hyperacusis underwent a four-month course of pharmacological therapy with bilateral sound generators (graded sound exposure) and CBT to target fear of sounds. Reduced hyperacusis-related symptoms were reported. Following this, the patient suspended treatment and underwent chemotherapy for breast cancer. Eight months later, after cancer treatment, the patient reported worsening of hyperacusis symptoms. Serotonin reuptake inhibitors and a further 4 months of CBT were prescribed. Following treatment, the patient reported an improvement in mood (depression, hostility, and sadness) and sound tolerance. In this case, no details were provided on the components of CBT. Hiller and Haerkötter [20] reported an RCT investigating CBT and the possible additional effects of sound stimulation on improving severity of tinnitus. In this study, one group received treatment comprised of an educational component (tinnitus education) in which patients learned about and applied psychological concepts such as “vicious cycle” and “coping cycle” to their personal situations with tinnitus. The second group completed ten sessions of CBT, including education (avoiding silence), changing thought processes (relaxation), diverting attention, and identifying avoidant behaviours and short/long terms consequences of behaviour. Half of each group were supplied with sound stimulation through behind-the-ear broadband white noise generators for each ear, with volume controls for graded increases. Following treatment, greater improvements in tinnitus severity were reported for patients with hyperacusis compared to those without hyperacusis. Notably, hyperacusis was not measured after treatment in this study.

### 3.8. Tinnitus Retraining Therapy

Sixteen studies explored the use of TRT for patients with hyperacusis as a primary complaint [21–25] or a secondary complaint [26–33] or as part of a symptom set [34–36] (Table 2.).

Nine studies [24–29, 32, 34, 35] focused on a classic TRT protocol to elevate hyperacusis with/without tinnitus. These studies incorporated a component of counselling including educational training in which the Jastreboff neurophysiological model is described to explain treatment and demystify the patients' experience. Guidance is given about avoidance behaviour (e.g., use of earplugs, avoiding environment sounds, or avoiding quiet) and the application of desensitising sound and sound enrichment was discussed. The depth of counselling and sound components depends on the treatment category (0–4: presence of tinnitus, hearing loss, hyperacusis, or noise exposure) assigned; for categories 1-2, sound generators are recommended; for categories 3-4 aimed at hyperacusis, bilateral open-fitting sound generators are fitted with instructions to gradually increase the sound daily to be tolerable without difficulties. Sound enrichment techniques are also used, in which digitally produced nature sounds are slowly reintroduced [24–29, 32, 34, 35]. Eight of these studies reported improvements in hyperacusis [25, 27, 29, 32, 35], three of which reported increased LDLs [24, 28, 34] following treatment. Hazell et al. [24] reported that, after 2 years of treatment, LDLs were well within the normal range (>100 dB) in over 60% of patients. In a case series, Formby and Gold [34] found that individual patients were reporting “noticeable subjective improvement in sound tolerance,” resolution of complaints of discomfort, and in one case “complete resolve of sound tolerance problems.” In a case report, Hesse et al. [35] described the need for the patient to build up a good therapeutic relationship before starting the sound exposure and generator component of therapy. This led to hyperacusis only occurring on very rare occasions. Both Suchova [29] and Molini et al. [27] reported that only a small number of patients with hyperacusis showed improvement following TRT. Molini et al. [27] reported that only one patient in hyperacusis category 3 achieved therapeutic success (a decrease of 2 or less on the scale of symptoms, a Tinnitus Handicap Inventory Grade 1, and an awareness of tinnitus value of less than or equal to 10% of the patient's wakefulness). In contrast, Forti et al. [26] found that TRT led to patients reporting no differences in difficulties with activities (relaxation, concentration, sleep, social relations, and work) following the treatment.

Three studies evaluated the effectiveness of using sound generators alongside directive counselling with patients reporting a primary complaint of hyperacusis [21], tinnitus [31], or hearing loss [30]. All three studies reported that LDLs had significantly improved over the course of treatment. In an RCT, Formby et al. [21] showed a clear pattern of LDLs initially increasing and then plateauing at 6 months after the onset of full treatment (counselling and noise generators). All three studies also reported greater treatment success and improvements of hyperacusis in patients who used noise generators in addition to directive counselling than those using part of the treatments (counselling alone, counselling and placebo noise generator, or noise generator only). In another RCT, Formby et al. [30] found that changes in judgements of uncomfortable loudness for the full treatment group after treatment averaged 15 dB–10 dB, compared to the changes of 5 dB or less in the other groups.

Gold et al. [22] investigated functional auditory changes demonstrated by increases in LDLs and DR during TRT for patients with tinnitus and hyperacusis. Following treatment, both LDLs and DR increased from the initial assessment whilst hearing threshold did not change significantly. The authors concluded that the DR can be increased following TRT. Patients self-reported an increase in quality of life and a decrease in the number of daily activities affected by tolerance problems. Similarly, Wölk and Seefeld [23] reported that regular use of maskers (set at hearing threshold and slowly increased) improved LDLs and DR and reduced severity of hyperacusis to “no longer a problem.” Formby and Keaser [33] explored the sound therapy component of TRT and potential treatment benefits for tinnitus patients using hearing aids (HA) versus audiometrically matched or LDL-matched tinnitus patients with hyperacusis using noise generators. Increases in LDLs and DR at follow-up were observed for both the audiometrically matched and LDL-matched groups. In the HA versus audiometrically matched noise generator condition, changes in hearing thresholds between groups were nominal, but patients using noise generators showed significant increments in LDLs compared to the HA group. In the HA versus LDL-matched noise generator condition, the changes in LDLs at 1 kHz were greater for the LDL-matched group than the HA group; differences were negligible at other frequencies.

In a case study of a patient with posttraumatic stress disorder reporting hyperacusis as a symptom, Westcott [36] reported that, following an initial treatment of antidepressants (a selective serotonin uptake inhibitor/a serotonin and noradrenaline reuptake inhibitor) for sound intolerance attributed to anxiety and depression, a TRT desensitisation program was carried out to help increase tolerance to environmental sounds. The patient self-reported an improved ability to cope with loud sounds, reduced reaction to unexpected loud sounds, and the ability to actively pursue her own piano playing.

One case report followed some of the TRT principles without explicitly stating this method [37]. Ruth and Hamill-Ruth [37] applied counselling with tinnitus habituation therapy (bilateral fitted in-the-ear-noise generators) to improve daily activities, relaxation, and mood monitoring in a patient experiencing both tinnitus and hyperacusis. In addition to therapy, the patient was prescribed Baclofen for pain and sleep disturbance. After one year, the patient no longer experienced tinnitus or hyperacusis and reported an improved ability to sleep and socialise.

### 3.9. Counselling Alone

Attri and Nagarkar [38] reported a case study using hyperacusis-focused directive counselling to educate the patient on the auditory system and mechanisms of hyperacusis (Table 2). Guidance and advice were given on avoiding silence, overprotection of ears, and use of background sounds to desensitise hyperacusis. The patient reported a reduction in HQ scores from 23 points (moderate) to 15 points (close to normal) and improved tolerance to sounds, with difficulties tending to occur only during depressive episodes.

### 3.10. Devices Alone

Eleven studies reported the effects of using different devices on patients with hyperacusis as a primary complaint or secondary complaint or as part of a symptom set (Table 3). Two studies [39, 40] reported “acoustic training” for hyperacusis which notably improved symptoms in the long term, with symptoms remaining in remission over a year later. These studies slightly differed in methodology. The seven “acoustic training” sessions (administered every 5 days) reported by Miani et al. [40] consisted of six to ten stimulations in which a narrow band noise was sent to an acoustic free field, before a 60 dB HL pure tone (other types of sounds were also used in later sessions) was sent through headphones for about 3 minutes followed by a pause of 3 minutes. The intensity of the sound stimulus and duration were increased in 5 dB HL steps (up to a total increase of 35 dB HL) and up to 5 minutes of exposure, until the last session, when the sound in the headphones reached 95 dB HL in all frequencies. High frequencies used in the sessions led to reports of discomfort by patients when listening via headphones, but not in the acoustic free environment. These sounds were not reflective of normal environmental sounds, so authors were not concerned. In contrast, Noreña and Chery-Croze [39] used passive exposure to an enriched acoustic environment (EAE). Sounds used were pure tones (based on the cut-off frequency of hearing loss) presented in random order for the duration of 100 ms with pauses between of 100 ms. Participants listened to the EAE through headphones for 1–3 hours a day at a just audible level. Auditory sensitivity significantly decreased at all frequency bands, from 2 weeks onwards. The impact of hyperacusis on activities and daily functioning was reduced, with all participants reporting significant decrease in both MASH and HQ scores over the course of the treatment.

Three studies reported desensitisation programs using broadband tinnitus maskers [41, 42] and pink noise tape cassettes [43]. To desensitise patients, sounds were presented in each ear through wearable tinnitus maskers or through headphones (for pink noise) and started at a threshold the patient could tolerate and gradually increased in loudness over the sessions. In the case series reported by Vernon [41], the patients wore ear plugs during the day and carried out the desensitisation program at night. All three studies reported improvements in hyperacusis, although some patients did not comply with the use of the tape cassettes as they were afraid to use them because they believed that it would aggravate their hyperacusis. One patient reported improved ability to manage life without hyperacusis limitations [42].

One RCT [47] evaluated the effectiveness of a continuous wave laser TinniTool versus a dummy laser device. A laser probe was inserted into a specifically designed headset which projects the laser beam (power output 5 mW) onto the tympanic membrane though a 17-degree diverging lens (creating a spot size of 1 cm) for 20 minutes a day, resulting in an energy density of around 6 J at the membrane. The placebo dummy device, apart from activation of the laser beam, reproduced all aspects of laser therapy. Tinnitus patients in the laser therapy group reported decreased hyperacusis. Seven patients in the laser therapy group and eight patients in the placebo group reported LDLs lower than 80 dB at baseline; of these, LDLs improved in five patients in laser therapy group compared to two patients in placebo group following treatment.

Two studies [44, 45] reported the long term effects of cochlear implants (CI) treatment in patients with unilateral hearing loss and tinnitus. Mertens et al. [45] found that hyperacusis only significantly reduced for the single-sided deafness group and not the asymmetric hearing loss group. Significant differences were observed in HQ total and the attentional subscale scores between the CI-ON and the CI-OFF condition for the single-sided deafness group. Ramos Macías et al. [44] reported that CI use resulted in improved sound tolerance for six out of seven patients, who showed a reduction in Sound Hypersensitivity Questionnaire scores [62]. Only one patient showed decreased sound tolerance problems. Saglier et al. [46] investigated the impact of family history of hearing impairment on rehabilitation using HAs and reported that hyperacusis scores were more improved in patients without a family history following fitting of HAs than those with a family history.

One study [48] reported that 21 sessions using a tinnitus progressive phase-shift treatment device (“Phase-outTM”), which presented a sound that resembles the frequency and amplitude of patients' own tinnitus but shifts 6° every 30 seconds, did not significantly change scores on the HQ or audiometric data between a pure tone tinnitus group and a narrow band noise tinnitus group. All measurements remained unchanged after therapy.

One case study [49] reported that diazide and methylprednisolone improved unilateral hearing loss but did not diminish hyperacusis. Following fitting of a custom-made binaural full-concha, unvented in-the-ear compression device (Micro Tech® Refuge-hyperacusic), the patient reported that the devices were very helpful and provided a level of protection that was at least as good as what he achieved with the plugs and muffs, but reported that he still could not carry out social activities (listening to music, attending concerts, restaurants, movies, and events, or hearing others when eating).

### 3.11. Pharmacological Therapy

Pharmacological therapy was reported in five case studies [50–54] where hyperacusis was part of a set of symptoms (Table 4). One case study [52] reports a patient placed on a diet to address metabolic factors, bisphosphonate and calcium, for otosclerosis. The patient reported feeling better until the treatment regime was discontinued, when symptoms of dizziness and hyperacusis returned. The patient was prescribed bisphosphonate risedronate and reported reduced complaints of symptoms and feeling best when the diet regime was followed. In another case study, Brookler [54] reported a patient prescribed metformin, risedronate (30 mg twice weekly), calcium, vitamin D, and sodium monofluorophosphate who reported worsening of hyperacusis on days when they took risedronate. Alternating the regime with etidronate (400 mg/day for 2 weeks) and risedronate (30 mg twice weekly for 4 weeks) did not improve hyperacusis but increasing risedronate intake to 30 mg twice weekly for 6 weeks and then for 11 weeks improved hyperacusis to be “almost gone.” Gopal et al. [51] reported differences in LDLs between conditions in which the patient was unmedicated and medicated with fluvoxamine (50 mg/day) and fluoxetine (20 mg/day), with improved LDLs above 100 dB in the medicated condition. Lee et al. [53] reported that antihypertensives and gabapentin (600 mg/day) reduced the intensity of both tinnitus and hyperacusis after 1 month. Nields et al. [50] reported that although six weeks of intravenous ceftriaxone led to a remission of “all symptoms,” excluding mild arthralgia and fatigue, hyperacusis subsequently worsened after treatment. Intravenous cefotaxim slightly diminished hyperacusis, but it remained an impediment to performing activities. Clonazepam led to a short-term lessening of hyperacusis symptoms but increased emotional lability. Following carbamazepine (blood level of 6 *μ*g/ml.), the patient reported that sound tolerance thresholds had increased, fear and irritability had lessened, and the kindling-like effects (sounds “adding up”) had diminished so she was able to recover more quickly.

### 3.12. Surgery

Six studies reported results from patients electing to undergo surgery specifically for hyperacusis [56, 58, 60], hearing loss and hyperacusis [57], intracranial aneurysms [55], or Meniere's disease [59] (Table 4). In a cohort study, J. Gavilán and C. Gavilán [59] performed a middle fossa vestibular neurectomy, which includes ablation of the vestibulofacial anastomosis, and removal of part of the nerve. They reported that hyperacusis symptoms had lessened or were no longer a problem. Nikkar-Esfahani et al. [56] reported that successful occlusion (permeatal blocking) of the round window surgery in two patients led to an improvement of conductive hyperacusis symptoms but caused a mild exacerbation of conductive hearing loss. Khalil et al. [55] reported a case study of a patient with hyperacusis whose magnetic resonance imaging (MRI) revealed a large aneurysm. A Guglielmi detachable coil (GDC) embolization of the aneurysm led to resolution of hyperacusis. Dang et al. [57] present a case of a patient with hearing loss and hyperacusis caused by bilateral superior semicircular canal dehiscence and posterior semicircular canal dehiscence. Surgery involved a right-sided transmastoid approach and temporalis fascia plugs of each defect. Hyperacusis was assessed as resolved at three months' follow-up. The patient did report residual imbalance and right ear fullness, however. Two of the most recent studies [58, 60] reported that transcranial round window niche and oval window reinforcement surgery led to improved hyperacusis, with increased LDLs and reduced Hyperacusis Questionnaire scores (author-developed questionnaire adapted from the GÜF [64]), but with no changes to hearing. One patient reported sustained improvements of hyperacusis and quality of life four years after surgery [58]. Silverstein et al. [60] reported higher success with unilateral surgery than bilateral surgery.

### 3.13. Future Research Priorities Identified in the Literature

Various further research priorities were identified in the included records. Gabriels [42] urged audiologists to pool their patient information together in order to study the link between symptoms of hyperacusis combined with having a limited DR. Most authors suggest that larger studies (Tables 2–4) need to be conducted to verify the effectiveness of CBT [18], TRT [28, 32], and different surgical treatments [56, 58, 60]. Studies evaluating the treatment benefit of counselling combined with alternative sound therapies (e.g., enriched acoustic therapy [39]) compared to those usually used in TRT should be conducted [30]. Larger studies focused on testing the effect of maskers/noise generators and HAs specifically for hyperacusis as a primary complaint are indicated [20, 21, 42]. Other possible treatments for hyperacusis suggested for further research include laser therapy [47], desensitisation techniques [41], and further investigation of the pharmaceutical treatments for certain subgroups of hyperacusis specifically when hyperacusis is induced by Lyme disease [50] or when cooccurring with depression [38]. Berry et al. [28] also highlight the importance of developing patient-based assessments for hyperacusis.

## 4. Discussion

This scoping review collated clinical research focused on management strategies used for hyperacusis, the definitions of hyperacusis, tools used for assessment and evaluation, and future research priorities. We found that more than half of the research currently reported was based on individual case studies and therefore cannot be generalised. In addition to this, management strategies were typically evaluated in patients reporting hyperacusis as a secondary complaint or as part of a symptom set, and as such the outcomes reported only provided an indication of effectiveness for hyperacusis. There is a lack of sufficient evidence to identify effective management strategies. These findings highlight an urgent need for controlled trials to evaluate the effectiveness of these management strategies for patients experiencing hyperacusis.

The definitions characterised by researchers had common terminology, with descriptions mainly differing in the emphasis placed on the direction of the sound tolerance, either reduction in tolerance or oversensitivity. The underlying theme reflected how the sound tolerance in patients with hyperacusis is different from “normal.” However, the appropriateness of “hypersensitivity” as a term for problems with sound tolerance has been questioned. Tyler et al. [12] argued that hypersensitivity reflects hearing thresholds that are better than normal and that hyperacusis is not usually associated with this and as such recommended avoiding using this terminology. The reference to “abnormal” could be upsetting to patients and lead to negative reactions to the experience. Although some studies referred to emotional reactions, such as distress and discomfort, and physical reactions of pain as characteristics of hyperacusis within their working definitions, the four distinct definitions (loudness, annoyance, fear, and pain) used in a recent narrative review [12] of hyperacusis were not readily used in the literature. The framework suggested by Tyler et al. [12] may be indicative of characteristics of hyperacusis rather than defining discrete subtypes that can be unambiguously differentiated. Consequently, there is a need for consensus through a systematic process involving professionals and patients to define hyperacusis and inform standards for assessment criteria.

To assess hyperacusis, most studies relied on self-report, with only some studies reporting use of a tool (LDLs, the HQ, or the TRT interview) to establish severity. Across the board, no consistent diagnostic tools or criteria were used, making it virtually impossible for comparisons across study populations. There is a need for established diagnostic criteria for hyperacusis and self-report measures appropriate for the population which have been evaluated for this purpose. The same can be said for outcome measures. The majority of studies did not report the outcome measures used. For case reports, the reliance was on clinical interview after treatment, which can be meaningful as the findings directly relate to patient experience. However, with the restricted information reported, we can only have limited confidence in the findings and the possible avenues for future research. Otherwise, a variety of outcome measures were reported, the most popular of which were LDLs and the HQ. Neither has been fully evaluated as outcome measure and in terms of the HQ was not designed to be used as an outcome measure. The three response options only provide coarse-grained categorical units of measurement and as such will not reliably detect small but potentially meaningful changes in hyperacusis [73, 74]. For LDLs, variability in the type of sounds used and the instructions given to patients' makes comparisons across studies relatively meaningless. There is an apparent need for clear guidance and consensus on LDLs use as outcome measures and for the development of self-report questionnaire specifically aimed at being responsive to changes in the impact of hyperacusis.

The most commonly reported management strategy was TRT, and most studies indicated that the treatment was beneficial to patients with hyperacusis. Complex interventions that include several interacting components require systematic evaluations [14]. Most included records reported here lacked rigorous methodology, raising concerns about the validity of the findings. Having said this, the RCTs using patients with a primary complaint [21] or secondary complaint of hyperacusis [30] did provide empirical evidence for the combination of the counselling and sound therapy components (full treatment) being of more benefit than the single components and as such highlighting an important principle of TRT. For the most part, there was a lack of rigor in reporting what was done in the name of TRT, particularly details of the counselling components used throughout the sessions or any differences when treating tinnitus and hyperacusis.

Despite the earliest research reported being published in the 1980s, there is an absence of research evidence on treatments directly aimed at treating hyperacusis. Most studies reported here were not focused on hyperacusis as the primary reason for management. This was especially true for the pharmacological treatments, all of which reported hyperacusis as part of a set of symptoms and were case studies; the effects observed may not be representative of the general hyperacusis population. The drugs reported were in general aimed at alleviating other symptoms and the reduction of hyperacusis may have been a byproduct of the reduction in those symptoms. Without the appropriate population, sample size, and systematic trial methodology, no strong conclusions can be reached.

Overall, only five of the 43 studies used a RCT design [18, 20, 21, 30, 47], two of which were solely aimed at patients experiencing hyperacusis [18, 21]. Whilst there are challenges in utilising a placebo control in interventions that involve sound therapy and/or counselling, waiting list controls designs could be implemented, as could a standard care versus specialist care design. The use of more robust trial designs in future hyperacusis studies would increase the quality and value of the evidence and support the development of optimised treatments.

## 5. Conclusions

Clear themes were identified from the definitions of hyperacusis reported, with an underlying theme that highlights the difference in sound tolerance from what is considered “normal.” In order to enable diagnosis and assessment, there needs to be an agreement on the definition including the perspectives of patients with lived experience of hyperacusis. The majority of management strategies were evaluated in patients reporting hyperacusis as a secondary complaint or as part of a symptom set and as such the outcomes only highlight potential benefits and no strong conclusions can be made. Authors agree that research should prioritise evaluating interventions and management strategies on patients with hyperacusis as a primary complaint, in particular TRT, sound therapy interventions, and pharmacological interventions. Importantly, to establish the benefits within this population, there is a need for controlled trials and appropriate patient-based assessments specifically for measuring hyperacusis. To date, only two controlled trials primarily aimed at hyperacusis have been conducted and as yet there are no registered ongoing or planned trials for hyperacusis as a primary complaint. With such an open field, it is essential that these opportunities for new research highlighted here lead to new controlled trials that will have a greater impact on the field.

## Figures and Tables

**Box 1:**
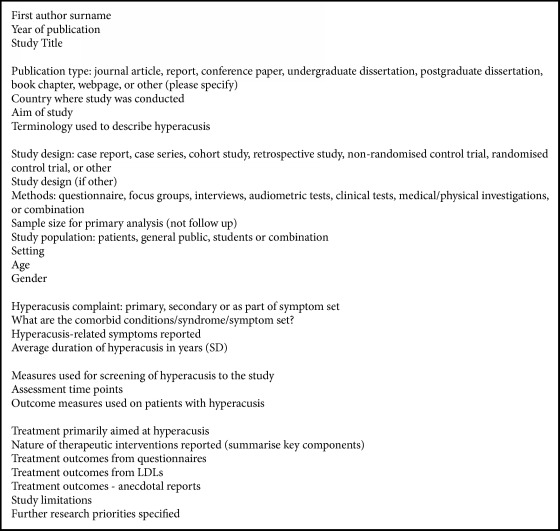
Data extraction fields.

**Table 1:** Search term strategies for hyperacusis interventions and outcome measures. CENTRAL = Cochrane Central Register of Controlled Trials; CINAHL = Cumulative Index to Nursing and Allied Health Literature; ISRCTN = International Standard Randomised Controlled Trial Number; ICTRP = the International Clinical Trials Registry Platform.

Search terms	Search engine
Hyperacus^*∗*^ AND [assess^*∗*^ OR measure^*∗*^ OR test^*∗*^ OR outcom^*∗*^ OR Diagnos^*∗*^ OR defin^*∗*^ OR treat^*∗*^ OR manag^*∗*^ OR therap^*∗*^ OR interv^*∗*^	Embase, PsycINFO, CENTRAL, Scopus, Web of Science
Hyperacus^*∗*^ AND [(assess^*∗*^ OR measure^*∗*^ OR test^*∗*^ OR diagnos^*∗*^ OR defin OR outcom^*∗*^ OR therap^*∗*^ OR interv^*∗*^ OR treat^*∗*^ OR manag^*∗*^)]	Web of Science, Scopus, PubMed, CENTRAL, CINAHL Plus
Hyperacus^*∗*^	Cochrane ENT Disorders Group Trials Register
Hyperacusis	ClinicalTrials.gov, ISRCTN, ICTRP
Hyperacusis AND [assessment OR measurement OR test OR Outcome OR diagnosis OR definition OR treatment OR Therapy OR intervention]	Google Scholar

**Table 2:** Charting population and outcome data according to management strategy: Cognitive Behavioural Therapy, Tinnitus Retraining Therapy, and Counselling. RCT = Randomised Controlled Trial; NRCT = Nonrandomised Controlled Trial; Retro = retrospective study; LDLs = Loudness Discomfort Levels; DR = Dynamic Range; HQ = Hyperacusis Questionnaire; TRT = Tinnitus Retraining Therapy; VAS = Visual Analogue Scale; HADS = Hospital Anxiety and Depression Scale; TSK = Tampa Scale of Kinesiophobia; QOLI = Quality of Life Inventory; TQ = Tinnitus Questionnaire.

Intervention	Ref	Study design	Sample size	Hyperacusis complaint	Aimed at treating hy	Outcome measures		Main findings
						Primary	Secondary	
CBT	Jüris et al. [18]	RCT	60	Primary	Yes	LDLs	HQ; HADS; TSK; QOLI	There were significant group effects for the treatment group (CBT) on all secondary outcome measures except for the anxiety subscale of the Hospital Anxiety and Depression Scale. There were both large significant between-group (treatment versus waiting list) and within-group effects for the CBT group on hyperacusis severity as measured by the HQ. LDLs were significantly reduced after treatment in CBT group compared to waiting list group for both ears.
CBT and pharma	Fioretti et al. [19]	Case report	1	Primary	Yes	HQ	LDLs, tonal audiometry	The patient reported improved mood and tolerance to sound with HQ scores below cut-off following second treatment. LDLs showed some improvement but were still below normal sound tolerance.
CBT and noise generators	Hiller and Haerkötter [20]	RCT	136	Secondary	No	Not stated, TQ	Not stated	At the 6-month follow-up, tinnitus education was shown to be more favourable. Improvements on TQ scores were observed for hyperacusis patients compared to all patients that completed the sessions. In the CBT group, similar results were shown with hyperacusis patients reporting improvements on TQ. More patients with hyperacusis than without were classified as responders for tinnitus education and CBT.
TRT	Formby et al. [21]	RCT	40	Primary	Yes	LDLs	Not stated	LDLs improved over the course of the intervention. These improvements were generally apparent within the first 4 months of the intervention, plateauing at around 6 months after the onset of full treatment (counselling and noise generators). Comfortable level speech scores improved from 50% to 80% after 6 months of full treatment. Treatment success rate was highest for the full treatment when compared to any of the partial treatments (counselling and placebo noise generator, noise generator only) or placebo noise generator group.
TRT	Gold et al. [22]	NRCT	130 (ears)	Primary	Yes	LDLs	Effect of hyperacusis on the patient's quality of life and daily activities questions	The patients self-reported an improved quality of life, with ability to comfortably participate in an increased number of activities after treatment. LDLs were significantly improved, at or near normal LDL thresholds for each frequency after treatment. Significant increases in DR at all test frequencies were also observed following treatment.
TRT	Wölk and Seefeld [23]	NRCT	122	Primary	Yes	LDLs, DR, Baltimore Questionnaire, VAS	Not specified	Patients reported that severity of hyperacusis was improved from a big problem to no longer a problem on the analogue scale. Regularly wearing maskers was associated with improved LDLs and DR, on average after treatment, indicating improved acceptance of ambient noise.
TRT	Hazell et al. [24]	Retro	187	Primary	Yes	LDLs, TRT questionnaire	Not specified	The number of life factors affected by hyperacusis was significantly reduced between the first and third visit after treatment. LDLs were significantly increased between each visit, with the majority of improvements experienced between the first and second visit. LDLs reached normal levels in 60% of patients by the fourth visit.
TRT	P. J. Jastreboff and M. M. Jastreboff [25]	Case series	201 (56 hy)	Primary/secondary	Yes	LDLs (not reported)	Not specified	LDL results were not presented. 56 patients reported hyperacusis (with or without misophonia); of these, 45 patients (80%) showed significant improvement after treatment. Improvement was higher for the hyperacusis and concurrent misophonia group (33/39) than hyperacusis alone patients (13/17).
TRT	Forti et al. [26]	Case series	40 (5 hy)	Secondary	No	Self-report	Not specified	No differences in difficulties with activities (relaxation, concentration, sleep, social relations, and work) were found in patients with hyperacusis following treatment.
TRT	Molini et al. [27]	Case series	81	Secondary	No	TRT interview	Not specified	64 patients (79%) from categories 0 and 4 improved and achieved therapeutic success at the end of 18 months based on a decrease in symptom scale score of 2 or less (focused on tinnitus). Only nine out of the ten achieved therapeutic success in category 3. One patient (out of 1) in category IV improved.
TRT	Berry et al. [28]	Cohort	32	Secondary	No	Subjective presence or absence of hyperacusis	LDLs	In the nine patients that presented with hyperacusis and tinnitus, LDLs were significantly improved following treatment.
TRT	Suchova [29]	Cohort	331	Secondary	No	Self-report	Not stated	Only 8 patients of 53 patients with hyperacusis showed an improvement in hyperacusis.
TRT	Formby et al. [30]	RCT	36	Secondary	No	LDLs for pure tone, speech, and white noise	Uncomfortably loudness judgements	All treatment groups showed improvements of LDLs (10 dB or more at two consecutive follow-ups). LDLs and loudness judgments in the full treatment group (counselling and sound therapy with binaural sound generators) were consistently greater (averaged 15 and 10 dB) than those measured for the other groups ((2) counselling and placebo sound generators, (3) binaural sound generators only, and (4) placebo sound generators). 82% of individuals were classified as successfully treated following full treatment, 50% following the neutral control treatment, and 25% and 40% following partial treatment.
TRT	McKinney et al. [31]	NRCT	182	Secondary	No	LDLs	Not specified	Incidence of hyperacusis in the treatment group was significantly lower and LDLs were significantly increased after 12 months. Improved LDLs were greater for those patients who used noise generators in addition to directive counselling or amplification. LDLs of patients who complained of specific sounds as being uncomfortable were not significantly different from the rest of the treatment group.
TRT	Bartnik et al. [32]	Retro	100 (40 hy)	Secondary	No	Author-developed questionnaire	Not specified	In category III, 55% of hyperacusis patients showed improvement (hyperacusis present, no prolonged noise exposure, and irrelevant or significant subjective hearing loss) and 60% in category IV (hyperacusis present, prolonged noise exposure, and irrelevant or significant subjective hearing loss)
TRT	Formby and Keaser [33]	Retro	51 (18 hy)	Secondary	No	LDLs, DRs	Not specified	No significant treatment changes for the audiometric thresholds. LDLs were significantly increased at each frequency following treatment. LDLs were significantly increased after treatment in the audiometrically matched hyperacusis noise generator treatment group compared to the hearing aid treatment group. LDLs between Hearing aid group and LDL-matched hyperacusis noise generator treatment groups for the 1000-Hz condition were significantly different.
TRT	Formby and Gold [34]	Case series	5	Within symptom set	No	LDLs	Not specified	In all five cases, LDLs were increased at 6 months, exceeding normal tolerance levels after a year of treatment. In one case, LDLs were improved as much as 25 dB after a 3-week period. Self-reported noticeable improvement in sound tolerance and complaints of discomfort to loud sounds had resolved.
TRT	Hesse et al. [35]	Case report	1	Within symptom set	Yes	Self-report	Not stated	The patient self-reporting hyperacusis rarely occurred after successful treatment.
TRT and pharma	Westcott [36]	Case report	1	Within symptom set	No	Self-report	LDLs (after treatment only)	No LDLs were measured at the beginning of treatment. LDLs were markedly lower than normal levels (>100 dB) after desensitisation treatment. Despite this, the patient self-reported that all sounds had become more tolerable, with an improved ability to tolerate environmental sound and to cope with loud voices and reduced reactions to impact of sound.
Counselling and tinnitus habituation therapy	Ruth and Hamill-Ruth [37]	Case report	1	Within symptom set	No	Self-report	Not stated	The patient no longer experienced severe tinnitus or hyperacusis and reported improved pain symptoms and ability to sleep and socialise one year after treatment.
Directive counselling	Attri and Nagarkar [38]	Case report	1	Primary	Yes	Hyperacusis test	LDLs, Hamilton psychiatric rating scale	The patient reported less difficulty with sounds. An improvement in hyperacusis test scores and LDLs indicating close to normal sound tolerance. The patient also self-reported suffering less depression and difficulty with sounds only occurring during depressive episodes.

**Table 3:** Charting population and outcome data according to management strategy: devices. RCT = Randomised Controlled Trial; NRCT = Nonrandomised Controlled Trial. Retro = retrospective study; LDLs = Loudness Discomfort Levels; LGOB = loudness growth in one-half octave band; MCL = Most Comfortable Loudness; HQ = Hyperacusis Questionnaire; SHQ = Sound Hypersensitivity Questionnaire; MASH = Multiple Activities Scale for Hyperacusis.

Intervention	Ref	Study design	Sample size	Hyperacusis complaint	Aimed at treating hy	Outcome measures		Main findings
						Primary	Secondary	
Acoustic training	Noreña and Chery-Croze [39]	Cohort	8	Primary	Yes	MASH, HQ, LGOB	LDLs	All participants reported a significant decrease in both MASH and HQ scores over the course of the treatment. Following the enriched acoustic environment treatment, auditory sensitivity significantly decreased at all frequency bands and all loudness categories (soft to too loud).
Acoustic training	Miani et al. [40]	Case report	1	Within symptom set	Yes	Discomfort levels	Not specified	The treatment produced notable improvement in the symptoms. No discomfort levels were registered in a free acoustic environment; discomfort levels were only observed with headphones at high frequencies that are not normally present in environmental noises. Clinical situation remained stable after 1 year.
Desensitisation program with tinnitus maskers	Vernon [41]	Case report	3	Primary	Yes	Self-report	Not stated	One patient was able to travel daily without ear protectors after 2 years of treatment. Another patient self-reported an improvement in condition after 3 months. Another reported improvement of at least 50% after 6 months of treatment.
Desensitisation program with tinnitus maskers	Gabriels [42]	Case series	2	Primary/within symptom set	Yes	MCLs and LDLs	Not specified	The patient self-reported noticeable improvements in hyperacusis with improved ability to manage life without the limitations associated with hyperacusis. LDLs and MCLs improved after four months. The patient reported ear plugs as being considerably beneficial.
Desensitisation program with tinnitus maskers	Hudson [43]	Case series	20	Primary	Yes	Self-report	Not stated	Patients self-reported improvements in hyperacusis after using tape. Some were afraid to use them.
Cochlear implant	Ramos Macías et al. [44]	Cohort	16	Within symptom set	No	SHQ	Not specified	Cochlear implant surgery resulted in improved sound intolerance, with a reduction in SHQ score for six patients (out of 7).
Cochlear implant	Mertens et al. [45]	Retro	23	Within symptom set	No	HQ	Not specified	Cochlear implants were found to significantly reduce hyperacusis for the single-sided deafness group, with significant differences observed in HQ total and attentional subscale scores between the cochlear implant ON and the cochlear implant OFF condition.
Hearing aids	Saglier et al. [46]	Cohort	177	Secondary	No	HQ	Not specified	Hyperacusis scores prior to fitting hardly differed between patients without a family history of hearing impairment and patients with family history. At 6 months after hearing aid fitting, patients without family history reported lower hyperacusis scores than those with a family history.
Wave laser TinniTool	Teggi et al. [47]	RCT	54	Within symptom set	No	LDLs	Not specified	Laser therapy led to a decrease in hyperacusis severity. LDLs for five patients (out of 7) improved in soft laser group, whilst only two patients in the dummy control group showed LDLs of more than 80 dB. Four patients presenting hyperacusis in the soft laser group also suffered from migraine compared to only 2 in the dummy group.
Phase-shift treatment device	Meeus et al. [48]	NRCT	21	Secondary	No	HQ	Not specified	No significant differences were found on hyperacusis questionnaire or audiometric data between pure tone tinnitus group and narrow band noise tinnitus group.
Suppression device and pharma	Valente et al. [49]	Case report	1	Within symptom set	Yes	Self-report	Not stated	The patient reported that the Micro Tech® Refuge-hyperacusic hearing instruments are very helpful and provided a level of protection that was at least as good as what he achieved with the plugs and muffs. The patient still reported an inability to listen to music, attend concerts, restaurants, movies, and events, or hear others when eating.

**Table 4:** Charting population and outcome data according to management strategy: pharmacological and surgery. LDLs = Loudness Discomfort Levels; HQ = Hyperacusis Questionnaire; GÜF = Geraüschüberempfindlichkeit.

Intervention	Ref	Study design	Sample size	Hyperacusis complaint	Aimed at treating hy	Outcome measures		Main findings
						Primary	Secondary	
Pharmacological	Nields et al. [50]	Case report	1	Within symptom set	No	Self-report	Not stated	Intravenous ceftriaxone led to a remission of all symptoms, but hyperacusis worsened afterwards. Intravenous cefotaxim diminished hyperacusis but did not completely abate it. Hyperacusis fluctuated. Clonazepam led to short-term attenuation of hyperacusis but increased emotional liability. Threshold for sound tolerance increased and sounds no longer seemed to “add up” following carbamazepine and patient reported increased ability to recover more quickly.
Pharmacological	Gopal et al. [51]	Case report	1	Within symptom set	No	(1) pure-tone threshold testing (250–8000 Hz), (2) impedance testing (tympanograms and acoustic reflex thresholds), (3) speech audiometry (speech threshold, word recognition score), (4) click evoked ABR testing, (5) transient evoked otoacoustic emission (TEOAE) testing, (6) uncomfortable loudness level (LDL) testing, (7) a screening test for central auditory processing disorders (SCAN-A testing), and (8) blood serum serotonin level		No remarkable clinical differences were reported by the patient between the unmedicated and medicated (fluvoxamine and fluoxetine) conditions for (1) pure-tone thresholds, (2) speech thresholds, (3) word recognition scores, or (4) tympanograms and (5) acoustic reflex thresholds. Only (6) LDLs were different between the two conditions, with the medicated condition showing increased sound tolerance.
Pharmacological	Brookler [52]	Case report	1	Within symptom set	No	Self-report	Not stated	Patient self-reported that following the diet regime made her feel better.
Pharmacological	Lee et al. [53]	Case report	1	Within symptom set	No	Self-report	Not stated	Hyperacusis and tinnitus persisted but intensity reduced after 1 month of treatment.
Pharmacological	Brookler [54]	Case report	1	Within Symptom set	No	Self-report	Not stated	The patient initially reported that hyperacusis was worse on risedronate. Following changes to the regime to risedronate, hyperacusis was less frequent and almost gone, although tinnitus and aural fullness remained present.
Surgery	Khalil et al. [55]	Case report	1	Primary	No	Self-report	Not stated	Hyperacusis completely resolved after procedure and had not returned after 10 months.
Surgery	Nikkar-Esfahani et al. [56]	Case series	2	Primary	Yes	Self-report	Not stated	Patients reported postoperative improvement in symptoms. Two years after the procedure, symptoms had continued to improve, with no significant problems.
Surgery	Dang et al. [57]	Case report	1	Primary	Yes	Self-report	Not stated	The patient self-reported that right-sided hyperacusis significantly improved at 1 month postop and completely resolved at 3 months postop. The patient was able to resume all choir-related activities.
Surgery	Silverstein et al. [58]	Case series	2	Within symptom set	Yes	HQ	LDLs (only postop data)	The patient reported marked improvement of hyperacusis and quality of life, with a decrease in hyperacusis survey scores and LDLs above 90 dB
Surgery	J. Gavilán and C. Gavilán [59]	Cohort	59	Within symptom set	No	Self-report	Not stated	In the majority of cases (63% = 37 cases), hyperacusis improved or disappeared following surgery. Only in two cases did hyperacusis become more severe, ten years after surgery.
Surgery	Silverstein et al. [60]	Cohort	6	Primary	Yes	Hyperacusis Questionnaire adapted from GÜF and LDLs	Not specified	All unilateral patients and two of the three bilateral patients reported subjective improvement in sound hypersensitivity, with reduced questionnaire scores and improved LDLs following treatment. The patients reported no change in hearing and improved quality of life after the procedure; one reported improved noise tolerance in social situations.
